# Circular RNAs: Key Regulators of Tumor Metabolic Reprogramming and Clinical Translation

**DOI:** 10.32604/or.2026.075012

**Published:** 2026-02-24

**Authors:** Yimao Wu, Yitong Liu, Ruowei Sun, Yiyuan Zhang, Qian Zhang, Chen Li, Mengyao Li

**Affiliations:** 1 State Key Laboratory of Systems Medicine for Cancer, Shanghai Cancer Institute, Renji Hospital, Shanghai Jiao Tong University School of Medicine, Shanghai, China; 2 Second Clinical Medical College, Guangdong Medical University, Zhanjiang, China; 3 Department of Basic Medical Sciences, Kangda College of Nanjing Medical University, Lianyungang, China; 4 College of Pharmacy, Inner Mongolia Medical University, Hohhot, China; 5 Department of Pharmacy, The First Affiliated Hospital of Guangxi Medical University, Nanning, China; 6 Shanghai Key Laboratory for Cancer Systems Regulation and Clinical Translation, Shanghai Jiading District Central Hospital, Shanghai, China

**Keywords:** Biomarkers, circRNAs, glutaminolysis, lipid metabolism, metabolic reprogramming, therapeutic targets, tumor metabolism

## Abstract

Tumor metabolic reprogramming is a core hallmark of cancer, characterized by pathways such as aerobic glycolysis, aberrant lipid metabolism, and glutaminolysis that support rapid proliferation and immunosuppressive microenvironments. Circular RNAs (circRNAs) are highly stable, evolutionarily conserved non-coding RNAs that have emerged as critical modulators of these metabolic shifts. This review aims to systematically elucidate the roles and mechanisms of circRNAs in reprogramming tumor metabolism, and to discuss their clinical potential as biomarkers and therapeutic targets. Through mechanisms including miRNA sponging, protein interactions, regulation of mitochondrial dynamics, and modulation of metabolic enzymes, circRNAs influence key metabolic pathways by targeting glycolytic enzymes, lipid synthesis regulators, and glutaminolysis-related molecules to either facilitate or inhibit their expression. This review systematically summarizes the unique contributions of circRNAs to tumor metabolic reprogramming, highlighting key mechanisms such as regulation of peptide-encoding protein translation, mitochondrial localization function, gene promoter-targeted transcriptional regulation, and cross-pathway metabolic mediation, which underscore their distinct biological advantages and regulatory roles in tumor metabolism. The stability and tissue specificity of circRNAs make them promising diagnostic biomarkers, while their role in drug resistance mediated by metabolic reprogramming highlights their potential as therapeutic targets. Strategies such as circRNA inhibitors, mimics, and nanoparticle-based delivery systems are being explored to modulate tumor metabolism. Despite challenges including complex regulatory networks and limited manipulation tools, advances in high-throughput technologies and clinical trials hold promise for translating circRNA research into novel cancer therapies.

## 1 Introduction

Tumor metabolism is a defining hallmark of cancer, characterized by metabolic reprogramming that includes the Warburg effect (a preference for aerobic glycolysis in cancer cells), as well as aberrant lipid metabolism, glutaminolysis, and mitochondrial dynamics [1,2]. This reprogramming not only provides cancer cells with the energy and biosynthetic precursors necessary for rapid proliferation but also contributes to the formation of an immunosuppressive tumor microenvironment (TME) [3,4].

Circular RNAs are a class of predominantly non-coding RNAs characterized by their covalently closed circular structures. These molecules exhibit high stability, resistance to nuclease degradation, and strong evolutionary conservation. They regulate gene expression through various mechanisms, including acting as miRNA sponges, binding proteins, or translating functional polypeptides [5,6]. The formation of circular RNA involves back-splicing reactions of exons or introns in pre-mRNA, primarily relying on base-pairing sequences present in the intronic sequences flanking the circularization region [7,8]. This pairing facilitates the binding of the 5^′^ splice site with the 3^′^ splice site, leading to the formation of a circular structure in the RNA molecule [9]. Based on their generation pathways, circular RNAs can be categorized into two major types: one type comprises circular transcripts produced from intracellular non-coding sequences post-processing, while the other is generated through a spliceosome-dependent processing method, where precursor mRNA (pre-mRNA) in eukaryotes undergoes splicing to yield circular RNA [10,11].

The metabolic patterns of cancer cells are significantly different from those of normal cells. The survival environment of tumor cells is often characterized by hypoxia, acidity, oxidative stress, and nutritional deficiency. In order to survive in this harsh microenvironment, tumor cells must regulate their own metabolic pathways to meet the needs of survival, a process of adaptive changes known as “metabolic reprogramming” in oncology [12]. In recent years, the relationship between RNA and tumor metabolic reprogramming has become an important research direction in the field of tumors. There is a close relationship between N^6^-methyladenosine (m^6^A) modification of RNA and the Warburg effect. It can regulate glycolysis through various mechanisms: stabilizing the mRNA of key glycolytic molecules (such as HK2, GLUT1) to activate the pathway; promoting the translation of related mRNAs (such as PDK4, MYC) to enhance the activity of related enzymes; regulating the degradation of negative regulators’ mRNA of metabolic pathways (such as APC), so as to promote the aerobic glycolysis reprogramming of cancer cells [13,14]. Recent studies have found that circRNA plays an important role in regulating tumor metabolism. For example, circRNA can target key enzymes (such as HK2 and GLUT1) or signaling pathways (such as PI3K/AKT) to promote or inhibit the Warburg effect [2,4]. Strikingly, these regulatory effects of circRNAs frequently converge on central metabolic regulators, including HIF-1α, MYC, p53, AMPK, and mTOR—key mediators that orchestrate metabolic reprogramming by integrating signaling cues with metabolic demands [15]. CircRNAs modulate the activity, stability, or transcriptional output of these core regulators thereby indirectly controlling downstream metabolic pathways and strengthening a functional link between circRNA networks and tumor metabolic plasticity [16,17]. Through such interactions with central regulators, circRNAs also impact lipid metabolism—for example, circEPB41 modulate lipid synthesis to drive tumor growth in liver cancer [18]. Moreover, certain circRNAs contribute to chemoresistance through regulation of metabolic pathways, including glycolytic changes associated with 5-FU resistance [19].

CircRNAs display distinct regulatory properties during tumor metabolic reprogramming, setting them apart from other non-coding RNAs. Their unique attributes primarily arise from their covalently closed circular structure and specific molecular mechanisms. In comparison to lncRNAs, circRNAs offer enhanced stability, resisting degradation by exonucleases and boasting a half-life roughly ten times longer than that of linear RNA. Moreover, they exhibit notably elevated expression levels in tumor tissues and associated body fluids [20]. Remarkably, circRNAs can act as “super sponges,” binding multiple metabolism-associated miRNAs to collaboratively modulate crucial metabolic pathways, including glycolysis and lipid synthesis [8].

The same circRNA may have different effects in different tumor cells. The function of circHIPK3 is significantly heterogeneous in various cancers, and this contradictory phenotype provides a key entry point for understanding its mechanism of action. In most cancers, such as breast cancer, colorectal cancer, lung cancer, etc., circHIPK3 is highly expressed by binding to tumor suppressor miRNAs such as miR-124-3p and miR-637, activating classic oncogenic pathways such as PI3K/Akt and Wnt/β-catenin, thereby promoting the proliferation, metastasis, and chemotherapy resistance of tumor cells and exerting pro-cancer effects. However, in bladder cancer, multiple studies have confirmed that the expression of circHIPK3 is downregulated, which can regulate the miR-588/HPSE axis and inhibit the autophagy pathway, showing anti-cancer properties. The core reason for this functional contradiction may be related to specific differences in the tissue microenvironment: the bladder tissue and tumor microenvironment contain high concentrations of hydrogen peroxide (H_2_O_2_), and existing evidence shows that H_2_O_2_ can directly induce the downregulation of circHIPK3 expression [21,22]. This tissue microenvironment-dependent functional switch suggests the functional heterogeneity of circHIPK3, and its clinical application needs to be accurately evaluated according to the type of tumor and the characteristics of the microenvironment [22]. This characteristic also brings complexity to the selection and determination of therapeutic targets.

The regulatory mechanisms of circRNAs in tumor metabolism are of great value. Their stability and tissue specificity make them promising biomarkers, as demonstrated by the use of exosomal circRNAs in urinary system tumors [23,24]. Targeting metabolically relevant circRNAs such as circPVT1 in triple-negative breast cancer (TNBC) may reverse abnormal metabolism and treatment resistance [25–27]. However, there remain critical knowledge gaps and challenges: the precise molecular cascade through which circRNAs coordinate cross-pathway metabolic regulation is not fully defined; the upstream signals that control functional switching of circRNAs in a tissue-specific manner have not been fully elucidated; and translational efforts to apply circRNA-based strategies in clinical practice are hindered by limited understanding of their long-term regulatory effects and optimal delivery paradigms.

This review aims to address these unresolved issues by systematically summarizing the molecular mechanisms of circRNA-mediated tumor metabolic reprogramming, dissecting the drivers of their functional heterogeneity, exploring their clinical potential as therapeutic targets or biomarkers for metabolism, and outlining critical future research directions to bridge the gap between basic research and clinical translation. By integrating recent advances, this article provides a theoretical framework to guide the development of targeted circRNA-based strategies for tumor metabolic intervention and discusses key barriers to their clinical application.

## 2 Tumor Metabolism: A Core Hallmark of Cancer

### 2.1 Overview of Metabolic Reprogramming

**Figure 1:**
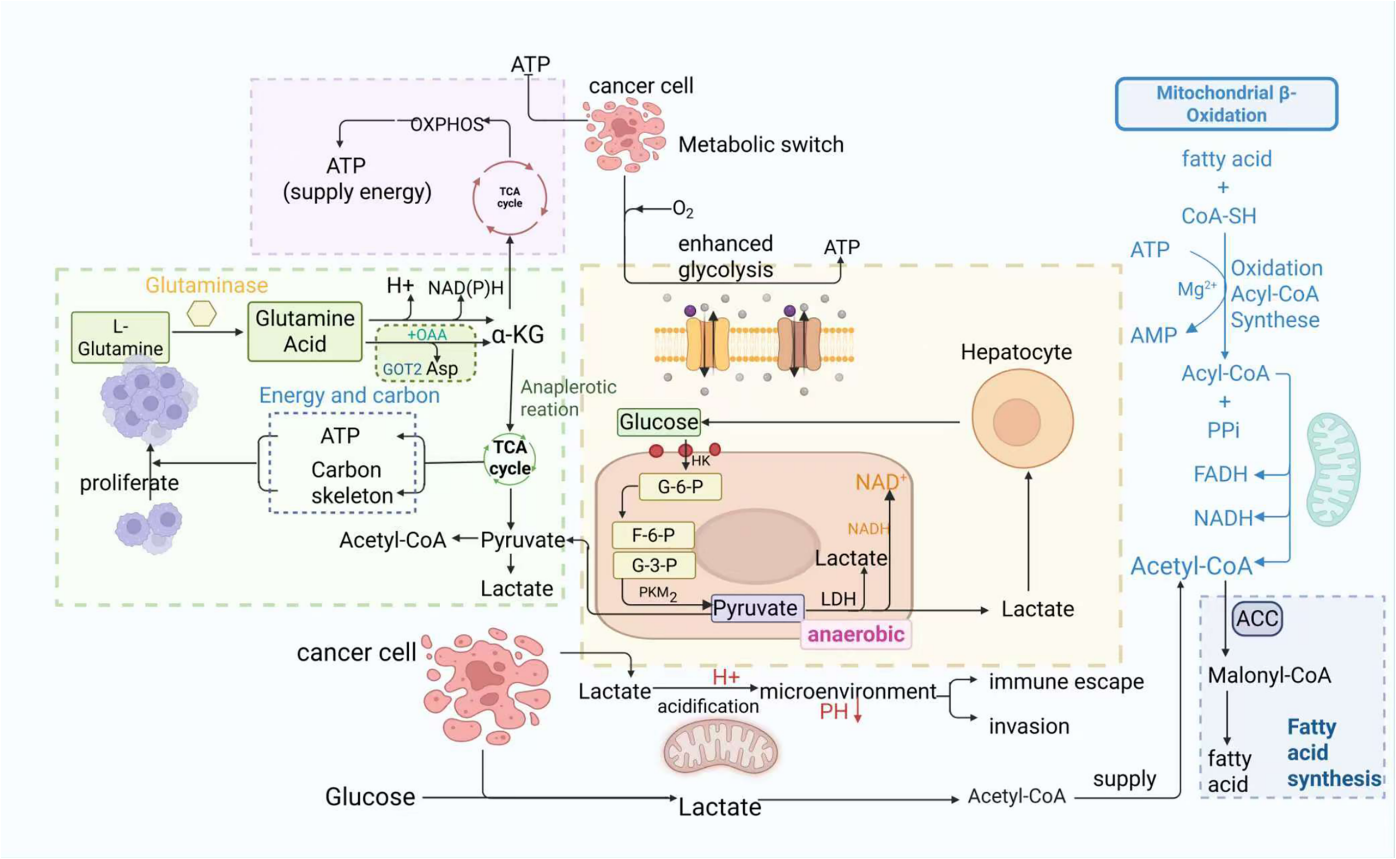
Schematic of metabolic reprogramming in cancer cells, highlighting key roles of glycolysis, glutaminolysis, and lipid metabolism, and lactate secretion’s impact on the tumor microenvironment. Glucose enters cancer cells and is converted to pyruvate via a series of enzymes, producing small amounts of ATP; unlike normal cells, this pyruvate is further converted to lactate by lactate dehydrogenase (LDH). L-glutamine is catalyzed by glutaminase to glutamate, which enters mitochondria as α-ketoglutarate (α-KG) to participate in the tricarboxylic acid (TCA) cycle, providing energy and carbon skeletons for tumor survival. Additionally, fatty acids and coenzyme A (CoA-SH) form acyl-CoA via acyl-CoA synthetase (consuming ATP), which enters mitochondria for oxidation, generating acetyl-CoA that feeds the TCA cycle and produces FADH_2_ and NADH—key for mitochondrial ATP production. Lactate secreted by cancer cells acidifies the tumor microenvironment, inhibiting immune cell function while promoting cancer cell proliferation and invasion, and also recycles acetyl-CoA to meet lipid precursor demands for membrane synthesis.

Metabolic reprogramming refers to the adaptive rewiring of bioenergetic pathways that tumor cells exhibit in order to survive and proliferate under harsh conditions such as hypoxia and nutrient deprivation [28]. As a key hallmark distinguishing them from normal cells, this reprogramming involves reshaped lipid metabolism, glycolysis, glutaminolysis, and mitochondrial dynamics. cancer cells take up glucose and produce lactate (aerobic glycolysis) even in the presence of oxygen, rely on glutamine for anaplerosis and biosynthesis (glutaminolysis), increase lipogenesis, and alter fatty acid oxidation [29,30]. These metabolic shifts not only provide energy but also support unlimited proliferation by providing essential precursors for biomacromolecule synthesis, which enables survival in nutrient-poor environments (Fig. 1) [29,30].

### 2.2 Key Metabolic Pathways in Reprogramming

#### 2.2.1 Aerobic Glycolysis (Warburg Effect)

The Warburg effect describes the preference of cancer cells to generate ATP via glycolysis followed by lactate production, even in the presence of functional mitochondrial respiratory chains and intact oxidative phosphorylation capacity [31]. This ‘glycolytic-dominant’ metabolism upregulates expression of glycolytic enzymes to meet energy demands and provide critical metabolic intermediates for biosynthesis, thereby promoting tumor growth and invasion [32,33]. The elevated levels of lactate from this process lower TME pH, impairing macrophage reprogramming and T cell function, thus suppressing antitumor immunity [34]. Targeting oncogenic signaling pathways that regulate this effect—such as MAPK/ERK and MAPK/JNK, which are involved in tumorigenesis beyond their normal role in sensing extracellular stimuli—has emerged as a promising anticancer strategy [35].

#### 2.2.2 Lipid Metabolic Reprogramming

Lipid metabolic reprogramming involves dysregulated (up- or down-regulation) processes of lipid uptake, desaturation, *de novo* synthesis, lipid droplet formation, and fatty acid oxidation in cancer cells [36]. Regulated by oncogenes, tumor suppressors, key transcription factors and growth factor signaling pathways [37], this reprogramming enhances lipid uptake, storage and synthesis to drive rapid tumor growth in various cancers [38]. Taking colorectal cancer cells as an example, tumor cells significantly increase their ability to uptake exogenous fatty acids by upregulating the expression of fatty acid transporters such as CD36 and FABPs. In CRC cells, the protein levels of key metabolic enzymes, including acetyl CoA carboxylase (ACC), fatty acid synthase (FASN) and stearoyl-CoA desaturase (SCD1) are significantly upregulated, thereby accelerating the *de novo* synthesis of endogenous fatty acids in tumor cells. The abnormally high expression of these metabolic enzymes not only provides sufficient precursors for lipid synthesis in tumor cells but also promotes tumor initiation and progression by activating multiple classic oncogenic signaling pathways such as Wnt/β-catenin and PI3K/AKT/mTOR [39,40]. Among them, the core metabolic enzyme FASN can enhance the proliferation and migration of colorectal cancer cells through AMPK/mTOR signaling pathway [41,42]. In addition to supporting tumor development via lipid acquisition and increased fatty acid oxidation, it also modulates immune cells to shape TME into a tumor-promoting niche [43]. For instance, enhanced lipid metabolism increases hyaluronan (HA) oligomers that facilitate cholesterol efflux in tumor-associated macrophages (TAMs) and drive their polarization toward M2 phenotype, thus accelerating tumor progression [44]. Such findings highlight the potential of therapeutically targeting TAMs to suppress their pro-tumor functions.

#### 2.2.3 Mitochondrial Dynamics

Mitochondria Dynamically undergo fusion and fission in response to environmental cues, a process known as mitochondrial dynamics [45] that is frequently deregulated in tumors and associated with drug resistance, cancer stemness, and metastatic potential [46–48]. Mitochondrial fission is mainly regulated by the cytoplasmic GTPase dynamin-related protein 1 (DRP1), which is recruited through a series of receptor proteins anchored on the outer mitochondrial membrane, including mitochondrial fission factor (MFF), mitofusin 49 and 51 kDa (MID49 and MID51) and fission protein 1 (FIS1). Studies have shown that different receptor proteins regulate distinct aspects of mitochondrial fission. Among them, MID49 and MID51 can bind acyl-CoA and act as sensors for β-oxidation metabolites, thus linking mitochondrial fission to changes in cellular metabolic status [49]. Fusion relies on mitofusins (MFN1/2) mediating outer membrane fusion and optic atrophy 1 (OPA1) mediating inner membrane fusion, whose core function is to maintain mitochondrial homogeneity and mtDNA integrity. When fission prevails, cells switch from oxidative phosphorylation (OXPHOS)-dependent metabolism to glycolysis; conversely, enhanced fusion favors OXPHOS [49,50]. The Increased activity of dynamin-related protein 1, a key regulator of fission, promotes mitochondrial fragmentation and increases metastatic capacity in breast, lung, and brain cancers [46]. Likewise, excessive fission drives hepatocellular carcinoma metastasis by regulating glucose metabolic reprogramming. Inhibitors of mitochondrial dynamics (mdivi-1) have demonstrated antitumor potential, underscoring the relevance of this pathway [51].

#### 2.2.4 Glutaminolysis

Glutaminolysis, the conversion of glutamine to glutamate and subsequently to α-ketoglutarate, is tightly coupled with mitochondrial dynamics [45]. Oncogene activation drives most cancer cells to rely heavily on the “glutamine anaplerosis” mechanism, shuttling glutamine-derived α-ketoglutarate into the tricarboxylic acid (TCA) cycle to fuel energy production [52,53]. As the most abundant circulating amino acid in blood and muscle, glutamine participates in multiple critical metabolic processes in cancer cells. Glutaminolysis also crosstalks with glycolysis, providing TCA cycle intermediates when glucose is limited [54]. Tumor cells, therefore, upregulate glutamine uptake and catabolism to support rapid proliferation [55], while glutamine also modulates intracellular signaling pathways to influence metabolic reprogramming [56]. The close interplay between the Warburg effect and glutaminolysis underscores their joint role in tumor progression, highlighting the therapeutic potential of targeting this crosstalk [53].

### 2.3 Drivers of Metabolic Reprogramming

#### 2.3.1 Genetic Factors

Metabolic reprogramming is driven primarily by genetic mutations in oncogenes and tumor suppressors [29]. Activated oncogenes, for example, RAS and BRAF mutations, induce a glycolytic preference and increased glutamine dependence. Unlike normal cells that reduce energy consumption during nutrient scarcity, cancer cells with RAS or BRAF mutations bypass such regulation to continue glucose uptake, ensuring sufficient energy for proliferation and survival [57]. Genetic mutation remains the central driver from initiation through metastasis, although the roles of metabolism and epigenetics have only recently been recognized [58].

#### 2.3.2 Epigenetic Regulation

**Figure 2:**
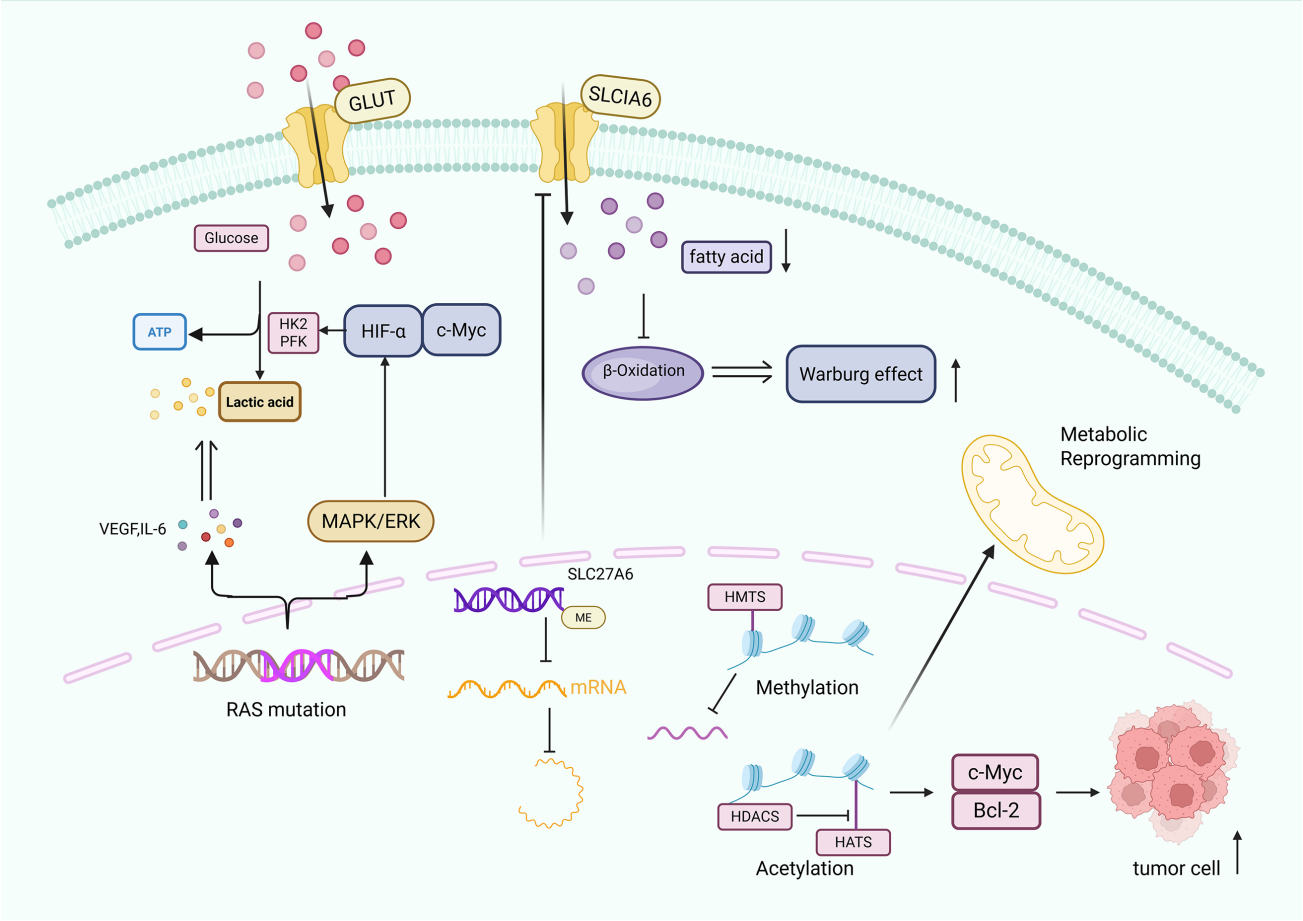
Molecular mechanisms driving tumor metabolic reprogramming: genetic mutations, DNA methylation, histone modifications, and their effects on glycolysis, lipid metabolism, and oncogene activation. Tumor metabolic reprogramming occurs via three main mechanisms: RAS mutations activate the mitogen-activated protein kinase/extracellular signal-regulated kinase (MAPK/ERK) pathway, upregulating glycolytic rate-limiting enzymes (hexokinase 2 (HK2), phosphofructokinase (PFK)) and prompting secretion of vascular endothelial growth factor (VEGF) and interleukin 6 (IL-6), thereby increasing glucose transporter (GLUT)-mediated glucose uptake and directing metabolism toward lactate production. DNA methylation of the solute carrier family 27 member 6 (SLC27A6) promoter downregulates its expression, reducing solute carrier family 1 member 6 (SLC1A6) (a related lipid transporter) to inhibit fatty acid β-oxidation and mitochondrial phosphorylation, shifting metabolism toward glycolysis. Histone methylation (regulated by histone methyltransferases (HMTs)) alters chromatin structure to repress tumor suppressors, while histone acetylation (regulated by histone deacetylases (HDACs) and histone acetyltransferases (HATs)) activates oncogenes like MYC proto-oncogene protein (c-Myc) and B-cell lymphoma 2 (Bcl-2), collectively driving metabolic reprogramming and abnormal proliferation. Additionally, hypoxia-inducible factor-α (HIF-α) is a key regulator in this process.

Epigenetic modifications, including DNA methylation, histone modification and chromatin remodelling, profoundly influence metabolic reprogramming [59]. Aberrant DNA methylation in tumor cells activates oncogenes and silences tumor suppressors and alters chromatin structure to rewire metabolic pathways. For example, hypermethylation of SLC27A6 downregulates its expression and promotes nasopharyngeal carcinoma (NPC) cell proliferation through altered lipid metabolism [60]. Histone modifications such as phosphorylation, acetylation, and methylation also have key roles. Histone methylation is mainly performed at lysine or arginine residues by histone methyltransferases (HMTs) using S-adenosylmethionine (SAM) as a cofactor [58]. Histone acetylation is regulated by histone acetyltransferases (HATs) and deacetylases (HDACs), which involves the HAT-mediated transfer of an acetyl group from acetyl-CoA to a histone lysine and is generally associated with transcriptional activation [61]. In addition to histones, acetylation directly modulates the function of proteins that are critical for oncogenesis; for instance, it can promote mitochondrial and upregulate glycolysis to drive metabolic reprogramming [62]. Furthermore, O-glcNAcylation of chromatin regulators changes chromatin morphology, further affecting gene expression. Together, epigenetic mechanisms regulate the expression of metabolic genes to modulate macromolecular synthesis and energy production, thereby orchestrating cancer cell reprogramming (Fig. 2) [63].

#### 2.3.3 Tumor Microenvironment

**Figure 3:**
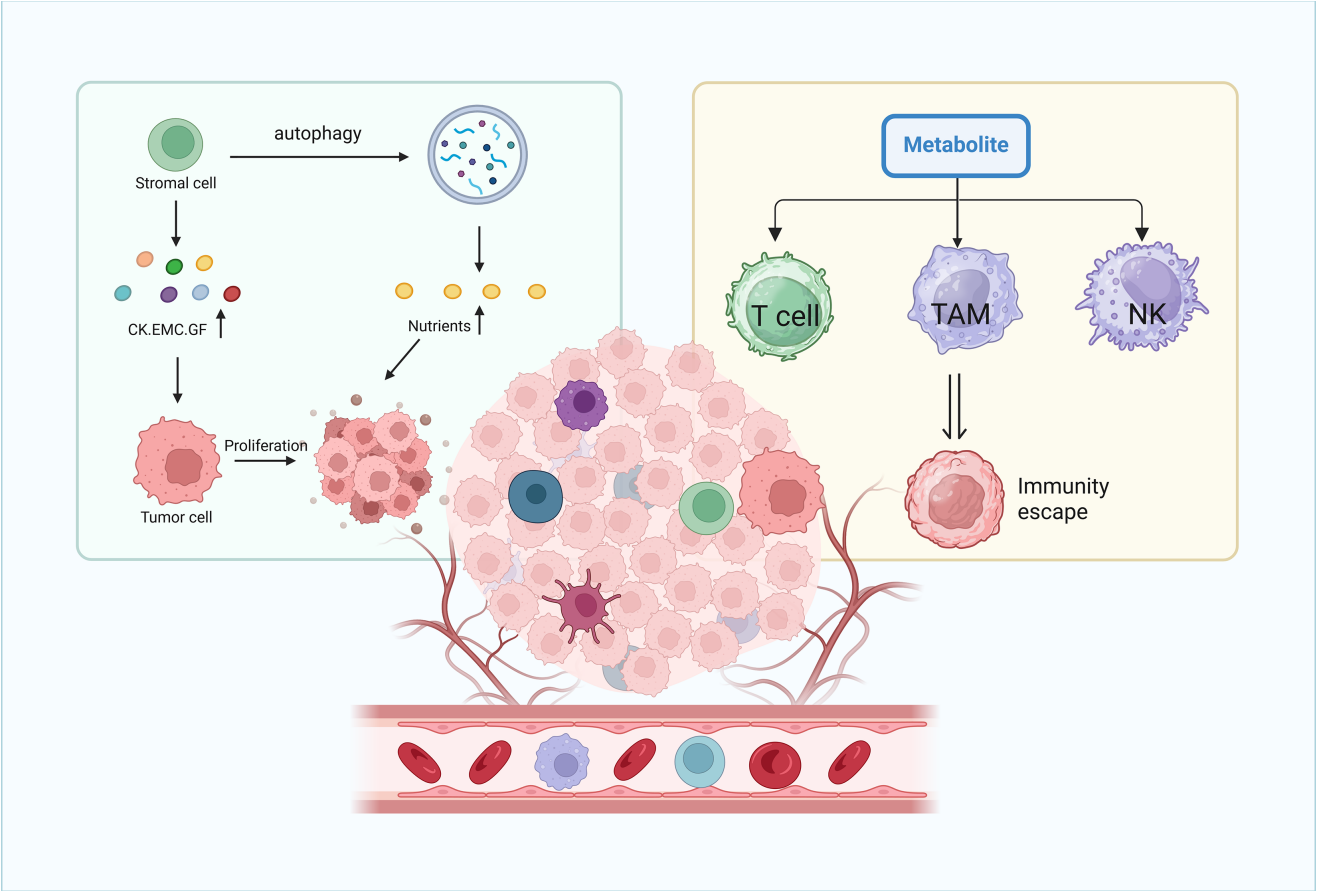
Role of the tumor microenvironment in supporting cancer cell proliferation and immune evasion through nutrient supply and immune cell modulation. The tumor microenvironment supports cancer cell proliferation and immune evasion: stromal cells release nutrients, cytokines (CK), extracellular matrix components (EMC), and growth factors (GF) via autophagy to promote tumor growth. Meanwhile, metabolites from cancer cell metabolism inhibit the function of T lymphocytes, macrophages, and natural killer cells, enabling tumor cells to evade immune recognition and elimination.

The TME, which is composed of fibroblasts, immune cells, and abundant stroma [64], shapes metabolic reprogramming by inducing scavenging mechanisms that force tumor cells to adapt to reduced nutrient availability [65,66]. For example, stromal cells secrete growth factors, cytokines and extracellular matrix (ECM) components that support tumor proliferation and invasion [67]; they also release amino acids via autophagy and pinocytosis to feed cancer cell metabolism [68]. Pancreatic cancer cells, for instance, use nucleosides and branched-chain keto acids produced by stromal cells under conditions of low glutamine [69,70]. Furthermore, metabolites derived from the TME modulate immune cell function, promoting an immunosuppressive niche that supports tumor progression (Fig. 3) [71–74].

In summary, metabolic reprogramming in tumor cells is a well-recognized hallmark of cancer, driven by genetic mutations, epigenetic modifications, and TME cues. These changes collectively supply energy and biomolecules to sustain tumor initiation, growth, and metastasis.

## 3 Roles of CircRNAs in Metabolic Pathways

### 3.1 CircRNAs and the Warburg Effect

Recent studies have highlighted that circRNAs are key molecules in metabolic reprogramming, which are deeply involved in the regulation of aerobic glycolysis in tumor cells through various mechanisms. It is worth noting that circRNAs play dual roles in tumor metabolism: some act as oncogenic regulators by promoting abnormal metabolic reprogramming, while others function as tumor suppressors by inhibiting dysregulated metabolic pathways. This functional duality not only complicates therapeutic targeting but also reflects an unresolved controversy in the field: whether the context-dependent effects of circRNAs arise from their intrinsic molecular properties or are predominantly shaped by cues from the tumor microenvironment, a distinction that remains poorly defined and necessitates integrative analysis of circRNA-interacting networks across heterogeneous tumor settings [75,76]. These mechanisms mainly include direct modulation of glycolytic enzyme expression, translation of functional polypeptides and miRNA sponge by competing endogenous RNA (ceRNA) mechanism. Competitive endogenous RNA (ceRNA) is a special class of RNA molecules, mainly including protein coding messenger RNA (mRNA), pseudogene, long non-coding RNA (lncRNA) and circular RNA (circRNA) [77]. These RNA molecules competitively bind to microRNA (miRNA) to exert their biological functions, thereby relieving the inhibitory effect of miRNA on its target mRNA [78]. In the occurrence and development of tumors, they are involved in regulating the proliferation, invasion and migration ability of tumor cells, and are closely related to drug resistance of tumors, and have important influence on the overall progress of tumors [79].

Direct regulation of glycolytic enzymes by specific RNA binding domains (RBDs) in the enzyme, such as hexokinase 2 (HK2), phosphofructokinase (PFK), and lactate dehydrogenase A (LDHA), represents a major pathway. For HK2, circ_0000376 has been shown to significantly upregulate HK2 expression through miR-577-mediated regulation of HK2 and LDHA, thereby promoting glycolysis and tumor growth in osteosarcoma [80]. Overexpression of circVMP1 increases Hexokinase domain component 1(HKDC1) levels by competitively binding with miR-3167, enhancing glycolysis and colorectal cancer progression [81]. In glioblastoma (GBM), U3 snoRNA-derived circRNA directly binds to the promoter of HK2 via transcription factor ZBTB7A, which enhances HK2 expression through the U3/ZBTB7A/HK2 regulatory axis to drive tumor progression [82]. For PFK, some circRNAs inhibit PFK activity to reduce aerobic glycolytic flux and shift cellular metabolism toward mitochondrial oxidative phosphorylation [15], while circ_0031242 positively regulates PFK expression through MAD2L1/miR-944 pathways, exerting antagonistic effects on glycolysis [83]. For LDHA, circRARS modulates its activity by binding to LDHA and enhancing its enzymatic activity to promote glycolysis in non-small cell lung cancer (NSCLC) [84], and the transcription factor YY1 can directly bind to the LDHA promoter through circRNA-mediated mechanisms to enhance its expression [10]. These diverse signaling pathways collectively regulate glycolytic enzyme expression, providing the potential to develop targeted therapies for specific cancer types. ENO1 is a key enzyme in the glycolytic pathway and circUBE2G1-99aa can bind to the ENO1 enzyme, regulating the activity of the ENO1/PI3K/AKT signaling pathway, inhibiting glycolysis, altering tumor metabolic patterns, thereby contributing to the inhibition of gastric cancer cell proliferation [85]. In addition, circRNAs affect tumor growth and proliferation by regulating key transporters involved in glycolysis. For example, ITCH has been shown to potentially suppress cancer cell proliferation by downregulating GLUT1 expression, thereby reducing glucose uptake in melanoma cells [86].

CircRNAs sponge miRNAs to alleviate the repression of glycolytic genes through a ceRNA mechanism. For instance, circ_0000376 sequesters miR-577 to relieve its inhibition on HK2 and LDHA, thereby activating the Warburg effect and promoting tumor cell glycolysis [80]. Similarly, circ_MAPK9 binds to miR-642b-3p to reverse its suppression of STAT3 and LDHA, leading to increased ATP and lactate production that supports tumor cell energy metabolism [87]. In gastrointestinal cancers, circRNAs fine-tune miRNA networks to regulate the expression of glycolytic proteins such as GLUT1 and HK2, highlighting their intricate roles in glucose metabolic regulation [88,89]. The covalently closed circular structure of circRNAs confers high stability, making them ideal diagnostic biomarkers [90]. These findings position circRNAs as critical nodes in glycolytic regulation with promising potential as targets for metabolic intervention. Notably, many circRNAs that regulate glycolysis also crosstalk with lipogenesis and glutaminolysis pathways, integrating multiple metabolic processes to support tumor progression. This interconnected regulation ensures coordinated metabolic rewiring, whereby circRNA-mediated modulation of glycolytic enzymes or signaling cascades simultaneously influences lipid synthesis and glutamine catabolism.

### 3.2 CircRNAs and Lipid Metabolism

CircRNAs regulate lipid metabolism through multiple mechanisms, with the ceRNA mechanism being particularly prominent. Importantly, circRNAs involved in lipid metabolic reprogramming often exhibit crosstalk with glycolytic pathways, as metabolic networks are highly interconnected in tumor cells. CircRNAs may target key molecules of both lipid synthesis and glycolysis simultaneously to ensure that energy production and biosynthesis are coordinated to meet the needs of the rapid proliferation of tumor cells [16]. By competitively binding miRNAs such as miR-24-3p, circRNAs release the suppression on key lipid synthesis pathways (PPARγ signaling pathway and Igf2/PI3K-AKT-mTOR) and thus upregulate fatty acid synthase expression [91]. In the liver, spatially specific regulation by circRNAs promotes lipogenesis in mitochondria of the central zone (cental zone, referring to centrilobular regions) and enhances inhibition of fatty acid β-oxidation in mitochondria of the periportal zone (periportal, referring to periportal regions) [92], dysregulation of this balance results in abnormal lipid accumulation [93,94].

In intrahepatic cholangiocarcinoma (ICC), elevated circMBOAT2 expression binds to PTBP1, facilitating the export of Fatty Acid Synthase (FASN) mRNA and promoting lipid metabolism [95]. In liver fluke-associated cholangiocarcinoma, circRNAs upregulate FASN, leading to free fatty acid accumulation and creating an immunosuppressive microenvironment; inhibiting FASN enhances the efficacy of PD-1 inhibitors [96]. The aberrant upregulation of circ-SLC9A6 in non-alcoholic fatty liver disease (NAFLD) intensifies hepatocellular lipid metabolic disorders, as evidenced by comparative studies on human and mouse liver tissues [97]. Furthermore, circPRKAA1 plays a role in maintaining lipid homeostasis by enhancing fatty acid synthesis and lipid storage. Its biogenesis is negatively modulated by AMPK activity, marking it as a pivotal element in lipid metabolic reprogramming [91]. Within cancer cells, circRNAs influence lipogenesis by modulating key enzymes such as fatty acid synthase (FASN) and acetyl-CoA carboxylase (ACC), thereby affecting lipid synthesis pathways [98].

### 3.3 CircRNAs and Glutaminolysis

CircRNAs modulate glutaminolysis through various molecular mechanisms, among which the ceRNA mechanism is well-characterized [99]. For instance, circ_0000808/miR-1827/SLC1A5 regulatory cascade sponges miR-1827 to release solute carrier family 1 member 5 (SLC1A5) (a glutamate transporter) from repression, thereby promoting glutamine uptake [100]. CircPVT1 competitively binds with miR-33a-5p to liberate c-MYC, which activates transcription of glutaminase (GLS) and accelerates conversion of glutamine into glutamate [25]. These circRNA-driven metabolic reprogramming events affect α-ketoglutarate production and energy supply in tumor cells, thus regulating carcinogenesis [101,102]. It should be noted that such regulation of glutaminolysis by circRNAs does not exist in isolation but is intertwined with glycolysis and lipogenesis, as tumor metabolism relies on the coordination of multiple pathways. CircRNA-mediated alterations in glutamine catabolism can modify the availability of metabolites that support glucose utilization and lipid synthesis, creating a synergistic metabolic environment conducive to tumor growth.

Under conditions of nutrient deprivation, circRNAs facilitate tumor cell survival and proliferation by modulating glutamine uptake and its subsequent conversion to glutamate. Certain circRNAs enhance the adaptability of tumor cells through the regulation of enzyme expression involved in glutamine metabolism, thereby promoting mitochondrial metabolic reprogramming [103]. Additionally, these circRNAs can impact oxidative phosphorylation and the TCA cycle, thus governing the utilization of energy and metabolic intermediates stemming from glutaminolysis [104,105]. This presents novel avenues for personalized cancer therapy.

### 3.4 CircRNAs in Mitochondrial Dynamics and Oxidative Phosphorylation

**Figure 4:**
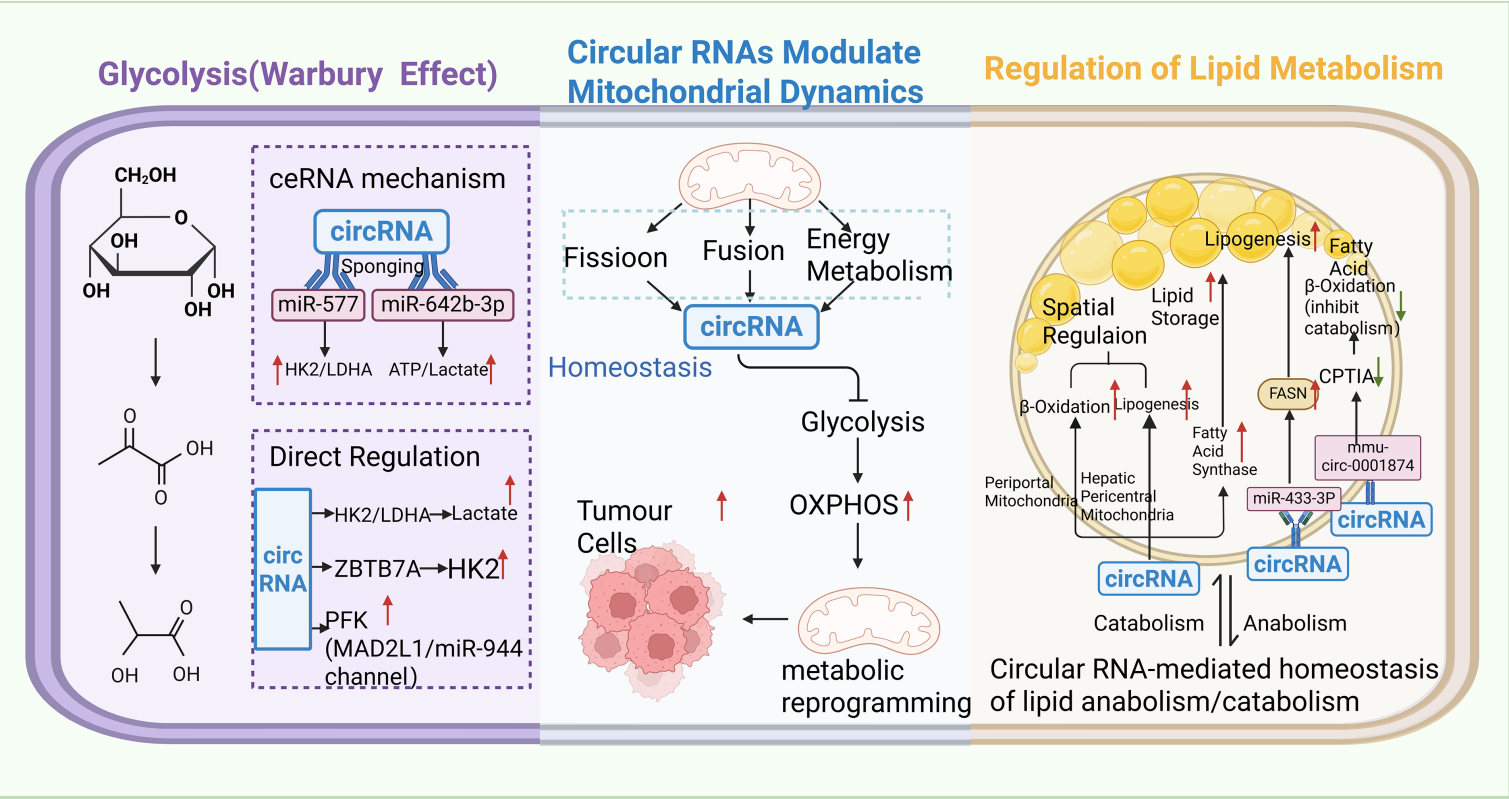
Mechanistic schematic of circRNA-mediated regulation of tumor metabolic reprogramming, encompassing glycolysis, lipid metabolism, and mitochondrial dynamics. CircRNAs regulate tumor metabolic reprogramming through three pathways: they act as competitive endogenous RNA (ceRNA) sponges for microRNA (miR) to affect hexokinase 2 (HK2)/lactate dehydrogenase A (LDHA), altering ATP and lactate levels, and directly bind enzymes to upregulate key glycolytic enzymes, promoting glucose conversion to pyruvate and then lactate to support glycolysis-dependent energy production. They also influence mitochondrial dynamics (fusion/fission) to regulate glycolysis and oxidative phosphorylation (OXPHOS), driving metabolic shifts. In lipid metabolism, circRNAs balance synthesis and degradation by spatially regulating mitochondrial lipid metabolism (pericentral vs. periportal regions), upregulating fatty acid synthase (FASN) to promote lipogenesis, and downregulating carnitine palmitoyltransferase 1A (CPT1A) to reduce fatty acid β-oxidation, meeting cancer cell growth demands. Additionally, genes such as zinc finger and BTB domain containing 7A (ZBTB7A) and mitotic arrest deficient 2 like 1 (MAD2L1) are involved in these regulatory networks.

CircRNAs are involved in mitochondrial dynamics and oxidative phosphorylation by regulating mitochondrial biogenesis, fusion/fission, and function of oxidative phosphorylation complexes, thereby affecting mitochondrial energy metabolism and cell proliferation [106,107]. For example, circMYLK4 inhibited glycolysis and promoted mitochondrial oxidative phosphorylation to regulate skeletal muscle energy metabolism via mechanisms involving voltage-gated channels and fatty acid β-oxidation in myofibers [108]. In cancer cells, circRNAs could promote mitochondrial metabolic reprogramming by modulating oxidative phosphorylation to support proliferation and survival [109,110]. Moreover, mitochondria-localized circRNAs have been implicated in the regulation of mitochondrial retrograde signaling that affects self-renewal of cancer stem cells. Key circRNAs involved in tumor metabolism regulation with their metabolic functions, targeted cancer types, and pathways are summarized in Table 1 and Fig. 4.

**Table 1:** Key circRNAs regulating tumor metabolism and their characteristics.

CircRNA Name	Metabolic Function	Cancer Type	Targeted Pathway	References
circMBOAT2	Binds to PTBP1	Intrahepatic cholangiocarcinoma	Lipid metabolism	[95]
circLARP1B	Reduces LKB1 protein levels	Hepatocellular carcinoma	Lipid metabolism	[111]
circACC1	Activates AMPK	Colon cancer	Lipid metabolism	[16]
circRIC8B	Downregulates LPL	Chronic lymphocytic leukemia	Lipid metabolism	[112]
circ_0069094	Promotes HK2 expression	Breast cancer	Glycolysis	[113]
circ_03955	Promotes HIF-1α expression	Pancreatic cancer	Glycolysis	[114]
cNEK6	Activates the mTORC1 pathway	Pancreatic ductal adenocarcinoma	Glycolysis	[115]
circNRIP1	Regulates the AKT1/mTOR axis	Gastric cancer	Glycolysis	[116]
Hsa_circ_00 07590	Reduces the stability of m6A-modified PTEN mRNA	Pancreatic ductal adenocarcinoma	Glycolysis	[117]
circECE1	Activates the c-Myc-TXNIP signaling pathway	Osteosarcoma	Glycolysis	[118]
circCDKN2B-AS1	Stabilizes HK2 mRNA	Cervical cancer	Glycolysis	[119]
circ-ENO1	Upregulates ENO1	Lung adenocarcinoma	Glycolysis	[120]
circLMO7	Upregulates GLS	Gastric cancer	Glutaminolysis	[121]

Note: PTBP1, Polypyrimidine tract binding protein 1; LKB1, Liver kinase B1; AMPK, Adenosine 5^′^-monophosphate-activated protein kinase; LPL, Lipoprotein lipase; HK2, Hexokinase 2; SKA2, Spindle and kinetochore associated complex subunit 2; HIF-1α, Hypoxia-inducible factor 1 alpha; mTORC1, Mammalian target of rapamycin complex 1; AKT1, RAC-alpha serine/threonine-protein kinase; mTOR, Mammalian target of rapamycin; m6A, N6-methyladenosine; PTEN, Phosphatase and tensin homolog; mRNA, Messenger RNA; c-Myc, MYC proto-oncogene protein; TXNIP, Thioredoxin interacting protein; HK2, Hexokinase 2; ENO1, Enolase 1; GLS, Glutaminase; mTOR, Mammalian target of rapamycin; LDHA, Lactate dehydrogenase A.

## 4 Targeting CircRNAs for Tumor Metabolic Therapy

### 4.1 CircRNA-Based Therapeutic Strategies

#### 4.1.1 CircRNA Inhibitors

Strategic approaches such as RNA interference (RNAi) technology, antisense oligonucleotides (ASOs), or the CRISPR-Cas system can silence oncogenic circular RNAs (circRNAs) through specific sequence recognition. RNAi tools like small interfering RNAs (siRNAs) and short hairpin RNAs (shRNAs) target circRNAs via complementary base pairing, incorporating the guide strand into the RNA-induced silencing complex (RISC) to facilitate the cleavage and downregulation of circRNAs [122]. To date, more than 20 siRNA-based therapies are in clinical trials for diseases including cancer [123]. In hepatocellular carcinoma, circRNA-SORE is upregulated in sorafenib-resistant cells; siRNAs targeting circRNA-SORE effectively silence this circRNA, significantly increasing cancer cell apoptosis and demonstrating therapeutic potential [124].

ASOs are another promising tool, consisting of unmodified or chemically modified single-stranded nucleic acid molecules (13–25 nt) that bind target RNAs through Watson–Crick base pairing to modulate their function [125]. *In vivo* studies have shown that ASO treatment inhibits tumor growth and alters the metabolism of tumor cells [126]. For example, ASO-circFOXK2 targets circFOXK2 in breast cancer cells and suppresses the growth of ER+ breast cancer *in vivo*. The antisense oligonucleotide (ASO-circFOXK2) downregulated circFOXK2 and reduced CCND1 expression at both mRNA and protein levels as well as p-RB and E2F target gene levels in BALB/C nude mice with MCF7 and drug-resistant MCF7 xenograft tumor models. This multitarget synergistic effect ultimately blocked the CCND1-CDK4/6 signaling pathway, thereby effectively inhibiting tumor cell proliferation and tumor growth [127].

**Figure 5:**
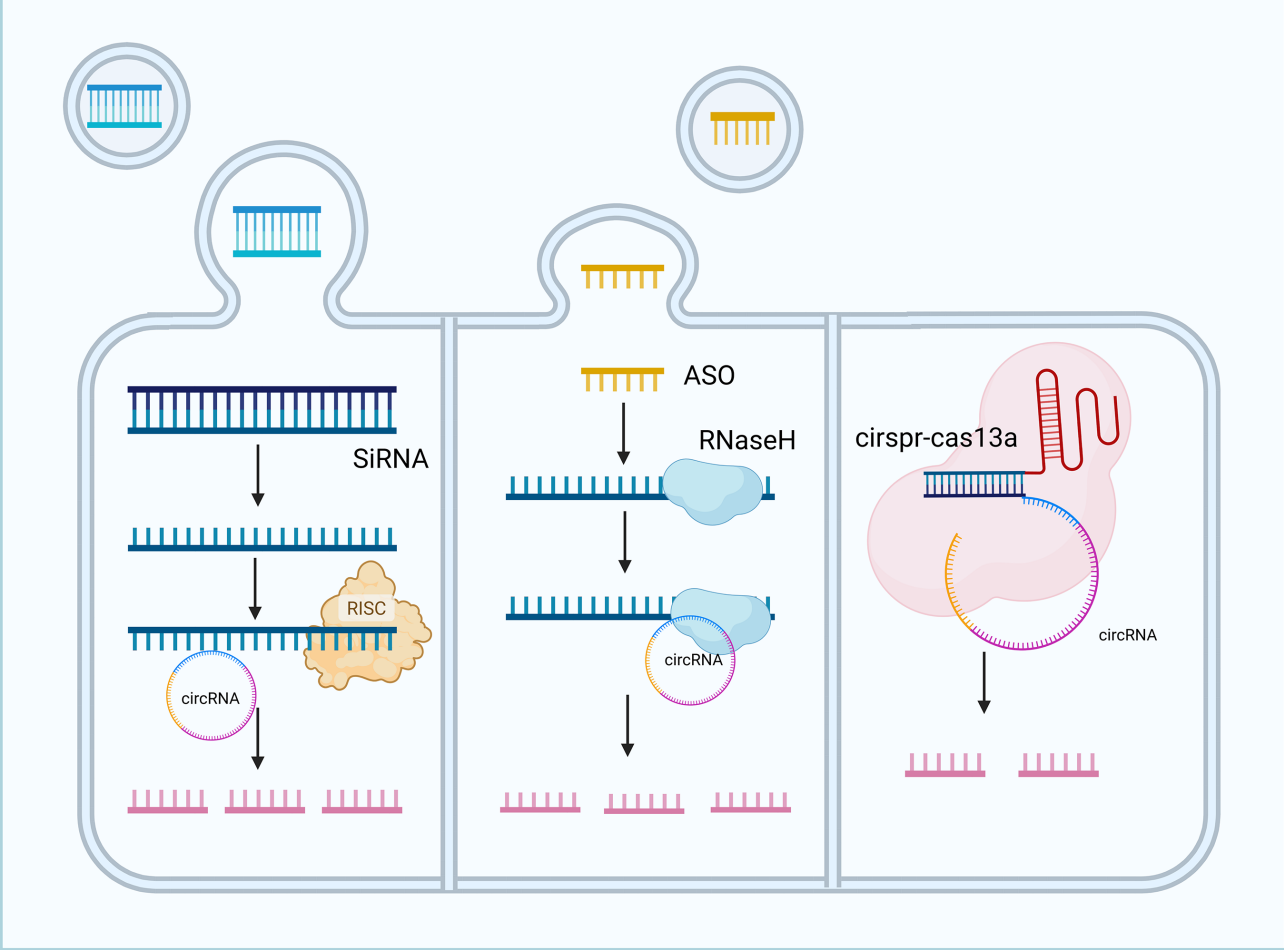
Strategies for targeting circular RNAs in tumor therapy. Small interfering RNA (siRNA)-mediated degradation, antisense oligonucleotide (ASO) mediated cleavage, and clustered regularly interspaced short palindromic repeats-CRISPR-associated protein 13a (CRISPR-Cas13a)-mediated disruption of circRNA functions. Three main strategies target circRNAs: siRNAs are delivered via lipid nanoparticles, bind endogenous RNA-induced silencing complex (RISC), and specifically degrade circRNAs to inhibit tumor-promoting activity. Antisense oligonucleotides form stable hybrids with circRNAs, recruiting ribonuclease H (RNaseH) to cleave circRNAs and block their functions. The CRISPR-Cas13a system uses guide RNAs to direct Cas13a to bind target circRNAs, activating its nuclease activity to degrade circRNAs and disrupt their regulatory roles in tumorigenesis and progression. siRNA, Small interfering RNA; RISC, RNA-induced silencing complex; ASO, Antisense oligonucleotide; RNaseH, Ribonuclease H; CRISPR-Cas13a, Clustered regularly interspaced short palindromic repeats-CRISPR-associated protein 13a.

While ASOs and siRNAs are widely used, they face challenges such as off-target effects. The CRISPR–Cas9 system provides a solution by efficiently targeting circRNA back-splice junctions and degrading partial or complete circRNA sequences without disrupting the function of linear coding mRNAs [128]. This revolutionary genome editing tool consists of an sgRNA and Cas9 nuclease that act as “molecular scissors”: the sgRNA–Cas9 complex recognizes PAM sequences near target DNA, guides Cas9 to induce double-strand breaks, and enables genome editing [129]. Innovations such as type II, III, and VI crRNP effector complexes have expanded CRISPR-based RNA targeting. Type VI Cas13–crRNA complexes cleaved target RNA in cis and non-target RNA in trans *in vitro* after binding to target RNA and undergoing conformational changes, with cleavage occurring outside the crRNA-binding region [130]. An Optimized sgRNA design for circRNA BSJ sites using CRISPR/Cas13d improved silencing specificity, which was validated through parallel screening of shRNA and CRISPR/Cas13d libraries [131]. Both long and short noncoding RNAs are now recognized as targets in precision oncology, offering robust approaches for cancer therapy (Fig. 5) [132].

#### 4.1.2 CircRNA Mimics

Restoration of tumor suppressive circRNAs can remodel abnormal metabolism. Tumor suppressive circRNAs function as miRNA sponges through the circRNA-miRNA-mRNA regulatory network, regulate linear RNA transcription by binding to promoters or transcription factors, or interact with proteins to inhibit tumor progression [133]. For example, in bladder cancer, circFNDC3B binds miR-1178-3p to suppress tumors [134]; hsa_circ_0072309 overexpression in breast cancer inhibited miR-492 activity and circASS1 acted via the miR-4443/ASS1 axis to impede progression [135]; circPABPC1 promoted non-ubiquitin proteasomal degradation of ITGB1 to inhibit metastasis in hepatocellular carcinoma [136]; circRNA ciRS-7 bound p53 to disrupt the p53/MDM2 complex and suppress gliomagenesis [137,138]. These findings highlight circRNAs as a focal point for cancer investigations, providing new insights into tumorigenesis [139].

The development of small molecules designed to specifically restore tumor-suppressive circRNA function or regulate their expression is a promising strategy for precision oncology, although the large size of circRNAs hinders cellular uptake and necessitates the use of nanoparticle carriers. Advancing our understanding of circRNA-metabolism axes will enable the delivery of artificially synthesized or engineered circRNAs into the TME to mimic natural tumor-suppressive functions, thereby offering precision and programmability in treatment. A combined approach utilizing both circRNA inhibitors and mimics—which silences oncogenic circRNAs while replenishing tumor-suppressive ones—could facilitate comprehensive metabolic regulation and has the potential to revolutionize personalized cancer therapy.

### 4.2 Drug Resistance and CircRNA-Mediated Metabolism

CircRNAs contribute to drug resistance through metabolic pathways, and targeting circRNA-metabolism interactions can overcome such resistance. Tumor drug resistance—where cancer cells evade chemotherapy, thereby reducing drug efficacy—poses a major barrier to treatment [126]. CircRNAs mediate resistance through mechanisms including acting as miRNA sponges, regulating signaling pathways, modulating cancer stem cell properties, promoting epithelial-mesenchymal transition [140], and also contribute to radioresistance via metabolic reprogramming in nasopharyngeal carcinoma [141], with distinct circRNAs either enhancing or suppressing resistance by altering drug sensitivity [142].

Metabolic pathways are key mediators of this resistance. In renal cell carcinoma, bull RNA sequencing identified circME1 as significantly upregulated in resistant cells [143]. ME1 is a multifunctional enzyme involved in aerobic glycolysis, NADPH production and lipid metabolism that can promote tumor drug resistance and is cis-regulated by circME [144]. CircME1 can upregulate the expression of ME1 to enhance aerobic glycolysis in clear cell renal cell carcinoma (ccRCC) and contribute to the development of its malignant phenotype [143]. These findings indicate that circME1 facilitates ccRCC resistance to sunitinib-targeted therapy through an ME1-dependent mechanism involving metabolic reprogramming [145–147]. Similarly, circABCC4 enhances cancer-associated fibroblast (CAF)-induced oxaliplatin resistance in pancreatic cancer via glycolytic reprogramming [148]. In CRC, oxaliplatin-resistant cells transfer exosomal ciRS-122 to promote glycolysis and resistance; in lung cancer, hsa_circ_0002130 regulates glycolysis-related genes to enhance osimertinib resistance [149,150].

Targeting circRNAs can reverse drug resistance. For example, downregulated in non-small cell lung cancer (NSCLC) tissues and cell lines, circ_0002483 sequesters miR-182-5p to target GRB2, FOXO1, and FOXO3, inhibiting NSCLC proliferation and sensitizing it to paclitaxel (PTX) [151]. In PTX-resistant ovarian cancer, hsa_circ_0002782 (circSLC39A8) acts as an endogenous sponge for miR-185-5p to target BMF, restoring PTX sensitivity [152]. CircFOXO3 also sensitizes NSCLC cells to cisplatin by regulating the miR-543/Foxo3 axis and promoting glycolysis [153].

Modulation of circRNA expression—via small molecules, ASOs or CRISPR/Cas9—can enhance chemosensitivity [154]. In breast cancer, ASO-circFOXK2 inhibited ER+ tumor growth in mice and restored tamoxifen sensitivity in resistant cells [127]. Nek2-targeting siRNAs and ASOs synergized with paclitaxel and doxorubicin to promote apoptosis, suggesting that combined antisense–drug therapy is a viable strategy [155].

### 4.3 Nanoparticle Delivery Systems

Nanoparticle-based delivery systems facilitate the efficient and targeted administration of circRNA therapies, with notable advancements in lipid nanoparticles, stimulus-responsive nanoparticles, gold nanoparticles, and exosomes [156,157].

Exosomes, extracellular vesicles measuring 50–150 nm with an average diameter of 100 nm, are ideal carriers owing to their stability, small size, and low toxicity [158]. Their lipid bilayer protects the cargo from degradation and facilitates transport to target cells [159]. *In vivo* and *in vitro* studies have demonstrated that exosome-delivered si-ciRS-122 reverses oxaliplatin resistance and glycolysis, thereby inhibiting CRC proliferation; RGD-exo-circDIDO1 suppresses gastric cancer progression without detectable toxicity [160,161].

Lipid nanoparticles (LNPs), spherical vesicles with a phospholipid bilayer that mimic cell membranes, are capable of encapsulating drugs and releasing small interfering RNAs (siRNAs) into the cytoplasm following endocytosis. These nanoparticles safeguard RNA interference (RNAi) molecules from degradation, augment cellular uptake, and mitigate immune activation to ensure effective delivery [162]. Both *in vitro* cell experiments and *in vivo* animal model studies have substantiated that the LNP-si circPDHK1 complex, when modified with the AS1411 aptamer, can efficiently deliver therapeutic si circPDHK1 into clear cell renal cell carcinoma (ccRCC) tumor cells, significantly downregulating the expression level of the target circPDHK1 [163].

Stimuli-responsive nanoparticles release cargo in response to tumor-specific enzymes, pH, glutathione, temperature, hypoxia or reactive oxygen species (ROS) and enable targeted drug accumulation in tumors with reduced off-target effects by enhancing specificity [164,165]. Gold nanoparticles targeting circDNMT1 inhibit tumor growth and deliver tumor suppressive circRNAs such as circFoxo3 to suppress melanoma [166]. In breast cancer models, gold nanoparticles directly target circDNMT1 or disrupt its RNA-binding protein (RBP) interactions [167]; silencing of circDNMT1 via specific siRNAs or ASOs that block its p53 and Auf binding sites significantly reduces *in vivo* tumor growth as circDNMT1 regulates autophagy through these interactions [168]. Specific examples of circRNA-targeting strategies and their corresponding delivery systems for various cancer types are outlined in Table 2.

**Table 2:** Targeting strategies of circRNAs and delivery systems in cancer.

Strategy	CircRNA Name	Cancer Type	Delivery Method	References
siRNA	circPDHK1	Clear cell renal cell carcinoma	Lipid nanoparticles	[163]
siRNA	circNRIP1	Gastric cancer	Organic cholesterol nanoparticles	[116]
siRNA	cPKM	Intrahepatic cholangiocarcinoma	Co-loading nanosystem	[181]
siRNA	circ_0058051	Hepatocellular carcinoma	Superparamagnetic iron oxide nanoparticles	[182]
siRNA	circPRMT5	Bladder cancer	SCNT nanoparticles	[183]
siRNA	circADARB1	Nasopharyngeal carcinoma	Semiconductor polymer nanoparticles	[141]
siRNA	CircDNMT1	Breast cancer	Gold nanoparticles	[184]
siRNA	circRHBDD1	Gastric cancer	PLGA-PEG nanoparticles	[185]

Note: siRNA, Small interfering RNA; circRNA, Circular RNA; PDHK1, Pyruvate dehydrogenase kinase 1; NRIP1, Nuclear receptor interacting protein 1; PKM, Pyruvate kinase M; PRMT5, Protein arginine methyltransferase 5; ADARB1, Adenosine deaminase RNA specific B1; DNMT1, DNA methyltransferase 1; ciRS-122, Circular RNA sponge for miR-122; RHBDD1, RH domain containing DEAD box protein 1; SCNT, Single-walled carbon nanotube; PLGA, Poly(lactic-co-glycolic acid); PEG, Polyethylene glycol.

To fully harness the therapeutic potential of nanoparticle systems, it is imperative to address challenges related to biodistribution, pharmacokinetics, long-term safety, and material synthesis [169]. By Optimizing nanoparticle properties and investigating combined delivery strategies, we can expedite their clinical translation.

Notably, long-term safety profiles exhibit significant variation across nanoparticle platforms: lipid nanoparticles (LNPs) demonstrate potential hepatotoxicity in preclinical models due to lipid accumulation in hepatic tissues, whereas exosomes display reduced immunogenicity stemming from their endogenous origin but encounter manufacturing challenges in achieving scalable production with consistent safety parameters [170–172]. A critical unresolved challenge in nanoparticle-mediated circRNA delivery involves reconciling targeted delivery efficacy with off-target immune activation; emerging evidence indicates that carrier material immunogenicity represents not merely an adverse effect but a potentially tunable parameter that could synergize with circRNA-mediated metabolic modulation, although the mechanistic links between carrier properties and immune-metabolic crosstalk remain incompletely characterized [173,174]. While Gold nanoparticles exhibit favorable biocompatibility in short-term applications, concerns persist regarding their chronic accumulation in organs such as the spleen and kidneys, which may precipitate oxidative stress or inflammatory responses during prolonged exposure [175]. Regarding immunogenicity, a central debate centers on the equilibrium between nanoparticle-induced immune stimulation and off-target inflammatory consequences: certain investigations reveal that surface-engineered nanoparticles can attenuate complement system activation and cytokine secretion, yet conflicting reports indicate that repeated dosing may elicit adaptive immune responses targeting the carrier materials themselves, thereby compromising therapeutic effectiveness [176,177]. Mechanistically, the immunogenicity of nanoparticle carriers is closely related to their size, surface charge and chemical composition—cationic nanoparticles are more likely to interact with negatively charged cell membranes and immune receptors, thus inducing higher levels of pro-inflammatory cytokines compared to neutral or anionic counterparts [178]. An emerging hypothesis from recent studies proposes that co-delivery of circRNAs with small molecule immune modulators could synergistically suppress undesirable immune responses while maintaining targeted delivery efficiency. To address long-term safety concerns, biodegradable nanoparticle materials are being developed to minimize organ accumulation, and patient-specific dosing regimens based on individual immune profiles may further reduce risks [179]. These advances underscore the need for rigorous long-term toxicological studies and translational models that mimic clinical administration schedules to ensure clinical viability of nanoparticle-based circRNA therapies [180].

## 5 Clinical Significance

### 5.1 Diagnostic Markers

CircRNAs have the potential to indicate metabolic dysregulation in specific cancers and are promising as diagnostic biomarkers. They can be detected in plasma and saliva [76], and their stability (half-life of approximately 48 h compared with ~10 h for mRNA) makes them useful. The fact that they are often expressed in a disease-specific, tissue-specific and tumor stage-specific manner allows precise diagnosis and even prognosis. Their presence in extracellular vesicles further enables noninvasive detection by blood tests, which supports early tumor identification [186].

In gastrointestinal cancers, exosomal circRNAs related to glycolysis are upregulated or downregulated in plasma and can be used as liquid biopsy markers for early detection. For instance, circPDK1 is significantly upregulated in the plasma exosomes and tumor tissues of pancreatic cancer (PC) patients and promotes cell proliferation and glycolysis *in vitro* and *in vivo*, demonstrating its value in distinguishing PC patients from healthy controls by non-invasive means [187,188]. For example, exosomal circPDK1 has entered phase I clinical trials for the diagnosis of pancreatic cancer [189]. Similarly, hsa_circ_0000181 is markedly downregulated in gastric cancer tissues and plasma samples, indicating that it may serve as a stable diagnostic marker [190]. Many studies have shown that some circRNAs exhibit better stability and diagnostic value than their corresponding mRNAs while reflecting the tumorigenic stage, highlighting their great potential in cancer diagnosis [140]. To improve the practical application of circRNA biomarkers in different types of cancer, key selection criteria include robust stability in body fluids, distinct tissue-specific and cancer-specific expression patterns, reliable detectability through non-invasive approaches, and consistent correlation with disease progression or treatment response.

### 5.2 Prognostic Markers

CircRNAs are associated with patient prognosis and tumor progression, making them valuable prognostic indicators. Their expression patterns can reflect cancer progression in clinical practice. For example, circ-LARP4 was downregulated in ovarian cancer (OC) tissues and significantly correlated with advanced FIGO stage, disease progression, and lymph node metastasis, positioning it as a potential prognostic marker for patients with OC [191]. Downregulation of circ_0009361 in CRC; its overexpression inhibited growth and metastasis, supporting its role as a prognostic biomarker [192]. In lung adenocarcinoma, upregulation of circPRKCI was associated with shorter overall survival in patients,thus identifying it as a poor prognostic factor [193]. A summary of the established diagnostic and prognostic values of circRNAs in various types of cancer is presented in Table 3.

**Table 3:** CircRNAs as diagnostic and prognostic markers in cancers.

CircRNA Name	Cancer Type	Diagnostic/Prognostic	References
hsa_circ_0000745	Gastric cancer	Diagnosis	[116]
hsa_circ_0013958	Lung adenocarcinoma	Diagnosis	[194]
circHIPK3	Lung cancer	Diagnosis	[195]
circZFR	CRC	Diagnosis	[196]
hsa_circ_101303	CRC	Diagnosis	[197]
circRNA-000911	Breast cancer	Prognosis	[198]
circBRAF	Glioma	Prognosis	[199]
circFOXP1	Gallbladder cancer	Prognosis	[200]
circ-ITCH	Bladder cancer	Prognosis	[201]
circPVT1	Bladder cancer	Prognosis	[202]
circRNA-104718	HCC	Prognosis	[203]
circUBAP2	Osteosarcoma	Prognosis	[204]
circVAPA	CRC	Prognosis	[205]
ciRS-7	CRC	Prognosis	[206]
hsa_circ_0007367	Pancreatic ductal adenocarcinoma	Prognosis	[207]
hsa_circ_0064428	Hepatocellular carcinoma	Prognosis	[208]
circSLIT2	Gastric cancer	Diagnosis and Prognosis	[209]

Note: hsa, *Homo sapiens*; HIPK3, Homeodomain-interacting protein kinase 3; ZFR, Zinc finger RNA binding protein; CRC, Colorectal cancer; BRAF, B-Raf proto-oncogene serine/threonine-protein kinase; FOXP1, Forkhead box P1; ITCH, Itchy E3 ubiquitin protein ligase; PVT1, Plasmacytoma variant translocation 1; HCC, Hepatocellular carcinoma; UBAP2, Ubiquitin associated protein 2; VAPA, Vesicle-associated membrane protein-associated protein A; ciRS-7, Circular RNA sponge for miR-7; SLIT2, Slit guidance ligand 2.

Early detection and intervention are crucial for improving clinical outcomes. Advancing techniques to detect circRNA-associated metabolic dysregulation may improve the sensitivity and specificity of early diagnosis. Large-scale clinical studies investigating the associations between circRNA expression profiles and prognosis, coupled with the development of accurate prognostic models, will provide a solid basis for clinical decision-making and patient management.

### 5.3 Therapeutic Applications

The integration of circRNA-targeted therapies with existing cancer treatment regimens holds significant promise [210]. Synthetic circRNAs have shown potential in protein expression, vaccine development, and RNA editing [211]. Non-protein/peptide-encoding synthetic circRNAs have been tested as therapeutic agents based on their physiological functions.

One application is to use synthetic circRNAs as miRNA or protein sponge. For example, a synthetic circRNA acting as a miR-21 sponge inhibited gastric cancer cell proliferation and downstream protein activation [212]. In gene editing, synthetic circRNAs can function as small interfering RNA (siRNA). dumbbell-shaped circRNAs consisting of two loops and a stem are specifically recognized and processed by the endoribonuclease Dicer into double-stranded siRNAs that mediate target mRNA interference [213]. Another design, circular siRNAs composed of circular sense RNA and its linear complementary RNA, enhanced gene silencing efficiency and reduced off-target effects compared with conventional siRNAs [214].

ASOs can target circRNAs by complementary pairing. Although seldom used to target back-splice junctions for circRNA knockdown due to their length, they efficiently block protein interaction sites on circRNAs [215]. Furthermore, the CRISPR/Cas13 system enables more specific circRNA knockdown than RNAi due to its low mismatch tolerance, although its *in vivo* efficiency remains to be determined [216].

CircRNAs also exert therapeutic effects through protein interactions. For example, circGSK3β promotes migration and invasion in esophageal squamous cell carcinoma (ESCC) by reducing β-catenin phosphorylation via decreasing GSK3β and its subsequent ubiquitination [217]. CircHuR inhibits gastric cancer (GC) proliferation, invasion and metastasis by sequestering CCHC-type zinc finger nucleic acid binding protein (CNBP) from the promoter of human antigen R (HuR), leading to downregulation of HuR expression [218].

Targeting circRNAs or their associated pathways, which have been implicated in oncogenesis, offers therapeutic potential. For instance, circHIAT1 and circATP2B1 are key regulators of RCC development, rendering their signaling pathways as promising targets for metastatic RCC therapy [219].

Protein-encoding synthetic circRNAs hold promise for applications in metabolic cancer vaccines [220]. While mRNA vaccines provide high immunogenicity and ease of production, their inherent instability and limited duration of protein expression compromise efficacy. Single-cell omics technology, through the analysis of the cellular heterogeneity of tumors and the immune microenvironment, offers vital insights for the design of personalized vaccine strategies targeting specific patient subgroups or the overcoming of immunosuppressive microenvironments [221]. Cyclization of linear RNA into circRNA improves stability, and encapsulation of antigen-encoding circRNA within lipid nanoparticles (LNPs) elicits potent innate and adaptive immune responses, thereby establishing a novel circRNA vaccine platform [222].

A recent study reported a circRNA-based vaccine for melanoma, which was synthesized *in vitro* using a type I intron-mediated PIE system and incorporated the CVB3 IRES element to facilitate translation [223]. Formulated with LNPs, this vaccine demonstrated superior therapeutic efficacy against “immune-excluded” MC38 and “immune-desert” B16 orthotopic tumor models in mice at one week post injection. Remarkably, the OVA-encoding melaoma circRNA vaccine also showed preventive effects by protecting naive hosts [142,224]. Although circRNA vaccines can induce dendritic cell maturation and activate both innate and adaptive immunity, further mechanistic studies and optimization are required to improve the suboptimal antibody response induced by CART-circOVA before it can be advanced as cancer immunotherapy [225,226].

Traditional mRNA vaccines have shown promise in tumor immunotherapy, yet they are hindered by inherent limitations such as poor stability, dependence on nucleotide modifications for functionality, and complex regulation of immunogenicity. In contrast, the distinct covalently closed-loop structure of circRNA vaccines, which lacks 5^′^/3^′^ termini, confers resistance to RNase degradation, resulting in superior stability compared to linear mRNA and notable longevity [173,227]. Furthermore, circRNA vaccines exhibit enhanced safety profiles, with no observed cytokine storm during immune activation, while effectively stimulating dendritic cell activity, robust antigen-specific CD8 T cell responses in lymph nodes and tissues, and potent antitumor effects, positioning them as promising therapeutic cancer vaccines [225]. Consequently, circRNA vaccines outperform traditional linear mRNA vaccines in terms of stability, translational efficiency, immune activation potential, and antitumor efficacy, offering a safer and more effective platform for tumor immunotherapy and potentially accelerating the clinical translation of RNA vaccines. Nonetheless, challenges remain, including low *in vitro* cyclization efficiency, potential interference from residual linear fragments, and unresolved concerns regarding clinical research, long-term human safety, and standardization of large-scale production processes [228,229].

## 6 Challenges and Future Directions

Despite the growing recognition of circRNAs in recent investigations, their study is confronted with formidable challenges, including complex mechanisms and limitations in manipulation.

CircRNAs regulate glucose and lipid metabolism through diverse mechanisms, including miRNA sponging, protein interactions, and protein translation, forming highly complex regulatory networks. they are also involved in cancer cell metabolism, and understanding these processes is crucial for identifying innovative therapeutic approaches [1,15,98]. For instance, circDDX21 is upregulated by transcription factors such as HIF-1α under glucose deprivation, influencing metabolic reprogramming in hepatocytes and driving carcinogenesis [230]. Notably, the mechanisms of circRNAs vary significantly across different metabolic diseases, necessitating tissue-specific analyses to clarify their roles and guide personalized therapeutic strategies, which adds complexity to cancer translational research [25,98,231,232]. To address this complexity, actionable research directions include integrating multi-omics data to map comprehensive circRNA-mRNA-miRNA-protein interaction networks, developing specialized bioinformatics tools to decode context-dependent regulatory patterns, and constructing dynamic computational models to simulate how circRNA-mediated metabolic regulation responds to cellular or environmental changes [233].

Furthermore, tools for precise circRNA manipulation are still limited and the controversy of how to reconcile the specificity of circRNA targeting with the pleiotropy of metabolic pathways remains a critical gap in knowledge that hinders translational progress. The lack of tissue-specific, spatiotemporally controllable editing systems not only prevents accurate manipulation but also raises questions about whether it is even possible to achieve sustained metabolic reprogramming by targeting individual circRNAs without disrupting physiological metabolic homeostasis [234]. The CRISPR/Cas9 gene editing system faces two core technical challenges: first, it remains significantly difficult to accurately predict off-target effects; second, editing efficiency is affected by multiple factors such as sgRNA design quality and delivery system selection. More importantly, there is an unavoidable tradeoff between reducing off-target activity and maintaining sufficient on-target cleavage efficiency [235,236].

Overcoming drug resistance is another critical challenge; although ASO delivery shows promise in reversing resistance, targeting circRNAs offers a complementary strategy to counter treatment resistance, such approaches are still in the early stages of clinical trials [237]. Although circRNAs hold potential as diagnostic and prognostic biomarkers [238], substantial improvements are needed in bioinformatics analysis of circRNA sequencing data, analytical precision, and subsequent precise regulation—underscoring the long and challenging path ahead in circRNA investigations.

Research on circRNAs is rapidly evolving with emerging future directions. One focus is the identification of metabolism-related circRNAs using high-throughput technologies. The integration of single-cell sequencing, spatial transcriptomics and multi-omics approaches can systematically dissect dynamic circRNA expression profiles in metabolic diseases [239,240]. For example, 164 differentially expressed circRNAs associated with obesity have been identified by high-throughput sequencing. In addition, artificial intelligence could predict circRNA-metabolic target interaction networks to accelerate biomarker discovery and has great potential for improving therapeutic efficacy and prognosis [241].

Another direction is to conduct clinical trials of circRNA-based metabolic therapy. This includes developing targeted strategies, such as circRNA inhibitors (for example, siRNAs or antisense oligonucleotides) or mimics (synthetic circRNAs), to regulate key metabolic genes (such as GLUT1 and HK2) [2,15,83]. In addition, combining nanodelivery technologies to explore their roles in regulating drug metabolism, therapeutic efficacy and toxicity profiles will improve the targeting and safety of circRNA therapy [237,242].

For clinical translation, it is advisable to prioritize the validation of circRNA therapeutics in metabolic cancers (for example, pancreatic and liver cancers) given that there are ongoing clinical trials targeting lipid metabolism pathways (for example, ANGPTL3/8 antibodies) [243–246], further investigation into the application of circRNA in CAR-T cell therapy—such as enhancing metabolic reprogramming through electroporation-mediated delivery of circRNA—demonstrates the potential of circRNA-based technologies to advance cellular therapies [242,247] (Table 4).

**Table 4:** Challenges and potential solutions in circRNA-based therapy.

Challenge	Description	Potential Solutions	References
Poor stability	Naked siRNA degrades rapidly in the extracellular environment	Develop novel delivery systems with precise targeting capabilities	[184,185]
Induction of immune response	Toxicity related to excessive cytokine release and inflammatory syndromes, which can induce high levels of inflammatory cytokines and interferons	Chemically modify siRNA, optimize sequences and structures, improve delivery systems, and reduce immune cell exposure	[248,249]
Off-target effects	Misrecognition by the RNA-induced silencing complex (RISC) and binding to other targets	Use CRISPR/Cas13 systems with low mismatch tolerance and design effective siRNA	[250,251]
Safety	Physiological toxicity of delivery lipids	Develop non-toxic targeted liposomes	[252]
Non-specific binding	Adverse effects on non-target tissues or cells	Develop therapeutic nanoparticle formulation methods	[253]
Insufficient cell or tissue penetration	RNA has difficulty penetrating cell membranes on its own.	Extend *in vivo* circulation, improve RNAi delivery specificity, and increase concentration to ensure effective penetration	[254]

Note: siRNA, Small interfering RNA; RISC, RNA-induced silencing complex; CRISPR/Cas13, Clustered regularly interspaced short palindromic repeats/CRISPR-associated protein 13; RNAi, RNA interference; *in vivo*, In the living organism.

## 7 Limitations

Although this review systematically summarizes the critical roles of circRNA in tumor metabolic reprogramming and its clinical translational potential, several limitations remain. Firstly, circRNA exhibits significant functional heterogeneity across different tumor types and tissue microenvironments, with its upstream regulatory signals and downstream effector networks not yet fully elucidated, posing challenges for precise intervention targeting specific circRNAs. Secondly, most current studies remain at the preclinical stage, where the efficacy and safety of circRNA as therapeutic targets or diagnostic biomarkers have not been adequately validated through large-scale clinical trials. Finally, existing circRNA manipulation tools still face deficiencies in specificity, delivery efficiency, and *in vivo* stability, while the long-term biosafety, immunogenicity, and targeting efficiency of nanodelivery systems require further comprehensive evaluation. Future research should integrate multi-omics data, develop more precise regulatory tools, and advance prospective clinical studies to facilitate the translation of circRNA from basic research toward practical applications in tumor metabolism therapy.

## 8 Conclusion

CircRNAs contribute to tumor metabolic reprogramming by modulating key pathways including glycolysis, lipid metabolism, and glutaminolysis. Their inherent stability and tissue-specific expression profiles establish them as promising diagnostic biomarkers for applications in early detection and disease monitoring, while demonstrating significant correlations with clinical outcomes such as chemotherapy resistance and tumor progression. As therapeutic targets, circRNAs present opportunities for metabolic modulation through silencing oncogenic circRNAs using siRNA, ASOs, or CRISPR-based tools, or restoring tumor-suppressive circRNA expression. Advancements in nanoparticle delivery systems have further expanded their translational potential. Despite existing delivery challenges, future research should prioritize resolving the context-dependent functionality of circRNAs and optimizing LNP-circRNA complexes for phase III trials in hepatocellular carcinoma. This review proposes A novel hypothesis that circRNA-mediated metabolic regulation could function as a “metabolic checkpoint” to selectively reprogram tumor metabolism without affecting normal cells, exploiting the unique stability and tissue specificity of circRNAs to target the interconnectedness of glycolysis, lipid metabolism, and glutaminolysis—a mechanistic insight with transformative potential for developing circRNA-based therapeutic strategies.

## Data Availability

Not applicable.

## Abbreviations

5-FU: 5-Fluorouracil
ACC: Acetyl-CoA carboxylase
ADARB1: Adenosine deaminase RNA specific B1
ANGPTL3/8: Angiopoietin-like 3/8
APC: Adenomatous polyposis coli
AUC: Area under the ROC curve
AS1411: Aptamer AS1411
ASOs: Antisense oligonucleotides
ATP: Adenosine triphosphate
B16: Mouse melanoma cell line
BET: Bromodomain and extra-terminal domain
BMF: Bcl-2 modifying factor
BRAF: B-Raf proto-oncogene
BSJ: Back-splice junction
CAFs: Cancer-associated fibroblasts
CAR-T: Chimeric antigen receptor T-cell
CCND1: Cyclin D1
ccRCC: Clear cell renal cell carcinoma
CD36: Cluster of differentiation 36
CDK4/6: Cyclin-dependent kinase 4/6
CDKN2B-AS1: CDKN2B antisense RNA 1
CREB5: cAMP-responsive element-binding protein 5
ciRS-7: Circular RNA sponge for miR-7
CNBP: CCHC-type zinc finger nucleic acid binding protein
circPVT1: Circular RNA plasmacytoma variant translocation 1
CoA-SH: Coenzyme A (thiol form)
CRISPR-Cas: Clustered regularly interspaced short palindromic repeats-CRISPR-associated protein
CRISPR/Cas13d: Clustered regularly interspaced short palindromic repeats/CAS13d
crRNP: CRISPR ribonucleoprotein
CRC: Colorectal cancer
CSCs: Cancer Stem Cells
DCR: Disease control rate
DFS: Disease-free survival
DLL3: Delta-like ligand 3
DRP1: Dynamin-related protein 1
DNMT1: DNA methyltransferase 1
ECM: Extracellular matrix
ECE1: Endothelin-converting enzyme 1
ENO1: Enolase 1
ESCC: Esophageal squamous cell carcinoma
FABPs: Fatty acid-binding proteins
FASN: Fatty acid synthase
FIGO: International Federation of Gynecology and Obstetrics
FISH: Fluorescence *in situ* hybridization
FIS1: Fission protein 1
FOXO3: Forkhead box O3
FOXP1: Forkhead box P1
FADH_2_: Flavin adenine dinucleotide hydrogen
GC: Gastric cancer
GBM: Glioblastoma
GEO: Gene Expression Omnibus
GLS: Glutaminase
GLUT1: Glucose transporter 1
GO: Gene Ontology
HA: Hyaluronan
HDACs: Histone deacetylases
HATs: Histone acetyltransferases
HIF-1α: Hypoxia-inducible factor 1 alpha
HIPK3: Homeodomain-interacting protein kinase 3
HK2: Hexokinase 2
HKDC1: Hexokinase domain component 1
HMGCS1: 3-Hydroxy-3-methylglutaryl-CoA synthase 1
HMTs: Histone methyltransferases
HuR: Human antigen R
IGF: Insulin-like growth factor
IGF2: Insulin-like growth factor 2
IGF2BP2: Insulin-like growth factor 2 mRNA-binding protein 2
IHC: Immunohistochemistry
IMP3: Insulin-like growth factor 2 mRNA-binding protein 3
ITCH: Itchy E3 ubiquitin protein ligase
KEGG: Kyoto Encyclopedia of Genes and Genomes
LDH: Lactate dehydrogenase
lncRNAs: Long non-coding RNAs
LMO7: LIM domain only 7
LNPs: Lipid nanoparticles
LXR: Liver X receptor
m^6^A: N^6^-methyladenosine
MAPK/ERK: Mitogen-activated protein kinase/Extracellular signal-regulated kinase
MAPK/JNK: Mitogen-activated protein kinase/c-Jun N-terminal kinase
MAD2L1: Mitotic arrest deficient 2 like 1
MC38: Mouse colon adenocarcinoma cell line
MFF: Mitochondrial fission factor
MID49/51: Mitofusin 49/51 kDa
MFN1/2: Mitofusins 1/2
mdivi-1: Mitochondrial division inhibitor 1
mtDNA: Mitochondrial DNA
mTOR: Mammalian target of rapamycin
mTORC1: Mammalian target of rapamycin complex 1
NADH: Nicotinamide adenine dinucleotide hydrogen
NAFLD: Non-alcoholic fatty liver disease
NPC: Nasopharyngeal carcinoma
NSCLC: Non-small cell lung cancer
OPA1: Optic atrophy 1
ORR: Objective response rate
OXPHOS: Oxidative phosphorylation
O-GlcNAcylation: O-linked β-N-acetylglucosamine modification
AMPK: Adenosine 5^′^-monophosphate-activated protein kinase
PI3K/AKT: Phosphatidylinositol 3-kinase/Protein kinase B
PD-1: Programmed death 1
PD-L1: Programmed death-ligand 1
PDK4: Pyruvate dehydrogenase kinase 4
PFK: Phosphofructokinase
PIE: Protein-intron-exon
PKLR: Pyruvate kinase, liver and RBC
PKM2: Pyruvate kinase M2
PPARs: Peroxisome proliferator-activated receptors
PPARγ: Peroxisome proliferator-activated receptor gamma
PPA2c: Pyruvate phosphate dikinase 2 cytoplasmic
PRMT5: Protein arginine methyltransferase 5
PTEN: Phosphatase and tensin homolog
PTBP1: Polypyrimidine tract binding protein 1
PTX: Paclitaxel
PFS: Progression-free survival
RAS: RAS oncogene
RHBDD1: RH domain containing DEAD box protein 1
RBP: RNA-binding protein
RNaseH: Ribonuclease H
RNAi: RNA interference
ROC: Receiver operating characteristic
RTKN2: Rhotekin 2
RISC: RNA-induced silencing complex
SAM: S-adenosylmethionine
SCD1: Stearoyl-CoA desaturase 1
SCNT: Single-walled carbon nanotube
sgRNA: Single-guide RNA
SLC1A5: Solute carrier family 1 member 5
SLIT2: Slit guidance ligand 2
SREBPs: Sterol regulatory element-binding proteins
SNRPA: Small nuclear ribonucleoprotein polypeptide A
siRNAs: Small interfering RNAs
shRNAs: Short hairpin RNAs
TAMs: Tumor-associated macrophages
TCA: Tricarboxylic acid cycle
TCGA: The Cancer Genome Atlas
TME: Tumor microenvironment
TNBC: Triple-negative breast cancer
TXNIP: Thioredoxin interacting protein
TXNDC5: Thioredoxin domain-containing protein 5
UBAP2: Ubiquitin associated protein 2
VAPA: Vesicle-associated membrane protein-associated protein A
Wnt/β-catenin: Wnt/β-catenin signaling pathway
YAP1: Yes-associated protein 1
YBX1: Y-box binding protein 1
ZBTB7A: Zinc finger and BTB domain containing 7A
ZFR: Zinc finger RNA binding protein
α-KG: α-Ketoglutarate
